# The Efficacy of *Moina micrura* Enriched with Probiotic *Bacillus pocheonensis* in Enhancing Survival and Disease Resistance of Red Hybrid Tilapia (*Oreochromis* spp.) Larvae

**DOI:** 10.3390/antibiotics10080989

**Published:** 2021-08-16

**Authors:** Nur Amalina Samat, Fatimah Md Yusoff, Nadiah Wan Rasdi, Murni Karim

**Affiliations:** 1 Laboratory of Aquatic Animal Health and Therapeutics, Institute of Biosciences, Universiti Putra Malaysia, Serdang 43400, Malaysia; gs51079@student.upm.edu.my; 2 Department of Aquaculture, Faculty of Agriculture, Universiti Putra Malaysia, Serdang 43400, Malaysia; fatimamy@upm.edu.my; 3 Department of Fisheries and Aquaculture, Faculty of Fisheries and Food Science, Universiti Malaysia Terengganu, Kuala Terengganu 21300, Malaysia; nadiah.rasdi@umt.edu.my; 4 Institute of Tropical Biodiversity and Sustainable Development, Universiti Malaysia Terengganu, Kuala Terengganu 21300, Malaysia; 5 Laboratory of Sustainable Aquaculture, International Institute of Aquaculture and Aquatic Sciences, Universiti Putra Malaysia, Port Dickson 71050, Malaysia

**Keywords:** live feed, *Moina micrura*, tilapia, enrichment, probiotic, *Streptococcus agalactiae*, streptococcosis infection

## Abstract

The administration of probiotics via live feeds, such as *Artemia* and rotifers, has gained significant attention. Moreover, indiscriminate use of antibiotics in conventional aquaculture practices in order to prevent or control disease outbreaks has resulted in the occurrence of residues and antimicrobial resistance. Thus, the application of eco-friendly feed additives, such as probiotics, as a safer alternative has received increasing attention in recent years. However, only minimal information on the administration of probiotics via freshwater cladoceran *Moina micrura* is available despite being commonly used for larval and post-larval feeding of freshwater crustaceans and fish. Thus, this study aimed to evaluate the application of *Bacillus pocheonensis* strain S2 administered via *M. micrura* to red hybrid tilapia (*Oreochromis* spp.) larvae. *Bacillus pocheonensis* that has been previously isolated from *Spirulina* sp. was subjected to preliminary in vitro evaluation of antagonistic properties. The agar well-diffusion assay revealed that this probiont could inhibit the growth of *Streptococcus agalactiae* and *Aeromonas hydrophila*. The size of inhibition zones ranged from 8.8 ± 0.2 to 18.2 ± 0.4 mm. *Moina micrura* was later used as a biological model in preliminary in vivo bacterial challenge assays to evaluate the efficacy of *B. pocheonensis* in protecting the host from diseases. *Moina micrura* was pre-enriched with *B. pocheonensis* at $10^{4}$ and $10^{6}\;\mathrm{CFU}\;{\mathrm{mL}}^{-1}$ before *S. agalactiae* and *A. hydrophila* were introduced into the culture. The study revealed that *B. pocheonensis* at $10^{4}\;\mathrm{CFU}\;{\mathrm{mL}}^{-1}$ was able to significantly enhance the survival of *M. micrura* after being challenged with both pathogens (63 ± 3%) in comparison to the control group. The relative percentage survival (RPS) of *M. micrura* was highest (*p* < 0.05) when treated with *B. pocheonensis* at both concentrations $10^{4}$ and $10^{6}\;\mathrm{CFU}\;{\mathrm{mL}}^{-1}$ (38.33) after being challenged against *S. agalactiae*. To assess the efficacy of *B. pocheonensis* in protecting red hybrid tilapia against streptococcosis, the larvae were fed with either unenriched (control) *Moina* or probiont-enriched *Moina* daily for 10 days. A significantly (*p* < 0.05) higher survival rate (77 ± 3%) was observed in larvae fed with probiont-enriched *M. micrura* compared to other treatments, and the RPS was recorded at 62.90. In addition, the *S. agalactiae* load was suppressed in larvae fed probiont-enriched *M. micrura* ($6.84\pm 0.39\;\mathrm{CFU}\;{\mathrm{mL}}^{-1}$) in comparison to the control group ($7.78\pm 0.09\;\mathrm{CFU}\;{\mathrm{mL}}^{-1}$), indicating that the probiont might have contributed to the improvement of tilapia health and survival. This study illustrated that *M. micrura* was suitable to be used as a vector for probiotics in freshwater fish larvae as an alternative to hazardous antibiotics for disease control.

## 1. Introduction

Despite the impact of the COVID-19 pandemic, global tilapia production grew by 3.3% in 2020, surpassing 6 million tonnes for the first time [1]. China remains the largest tilapia producer at 1.62 million tonnes, accounting for over a quarter of the total global production in 2018 [2]. Although the domestic sales of tilapia products have recovered since the Chinese economy reopened in late March 2020 after the easing of COVID-19 lockdown measures, the exports of tilapia products have been drastically affected [3]. In Malaysia, tilapia is the second most reared freshwater fish after the African catfish (*Claris gariepinus*), contributing to about 30.7% of the total freshwater aquaculture production in 2018 [4]. The red hybrid tilapia is preferably cultured by fish farmers, accounting for more than 94% of the total tilapia production [4]. The Department of Fisheries (DOF) in Malaysia has established 28,099 ha of aquaculture industrial zone (AIZ) for a high impact project (HIP) across the country involving marine fish and shrimp farming, freshwater fish farming, cockle farming, seaweed farming, ornamental fish production, and hatcheries [5]. The Como River, flowing into Kenyir Lake, Terengganu, Malaysia is a large-scale AIZ with 2800 ha of total land dedicated to tilapia farming [6].

Despite the increasing demand for fish as a healthy source of animal protein, the aquaculture industry has always been hampered by disease outbreaks, causing a significant economic loss, especially in developing countries [7,8]. Although tilapia is considered capable of tolerating a broad range of environmental conditions, over half (54.9%) of reported disease cases in tilapia culture were caused by pathogenic bacteria [9]. As a consequence of the intensifications of cage aquaculture activities around the Como River, mass mortality of cage-cultured red hybrid tilapia was reported in February 2012 [10]. In addition, mortality outbreaks of cage-cultured red hybrid tilapia in Kenyir Lake, Terengganu and Pergau Lake, Kelantan, Malaysia during the dry season from March until June have been periodically reported since 2000 [11]. Detection of *S. agalactiae* in the sampled organs suggested the possible case of streptococcosis associated with warm water [11]. Recently, in January 2020, a tilapia farm in Selangor, Malaysia reported 70% mortality of adult red hybrid tilapia whereby co-infections by tilapia lake virus, *A. hydrophila*, and *S. agalactiae* was identified as the main cause [12].

In Malaysia, streptococcal infection is primarily controlled by incorporating antibiotics, such as erythromycin and oxytetracyclines, into feed [13,14]. However, the rapid emergence of antibiotic-resistant pathogens is a primary concern worldwide, threatening the efficacy of antibiotics. It has been reported that *S. agalactiae* isolated from commercial red hybrid tilapia farms in four states in Malaysia was resistant to spiramycin, olendomycin, sulphamethoxazole, oxolinic acid, kanamycin, and nalidixic acid [13]. Moreover, antibiotics in cultured fish in Malaysia have never been extensively monitored [15]. The application of probiotics in aquaculture practices has been extensively studied as a safer alternative to antibiotics [16,17,18]. In general, probiotics are defined as ‘live microorganisms which when administered in adequate amounts confer a health benefit on the host’ [19].

Probiotics can be introduced into the host via feeding probiotic-supplemented pellet food, feeding on probiotic-enriched live feeds, direct addition to the water column, or injection [20]. Encapsulating probiotics in live feeds, such as *Artemia* and rotifers, is gaining popularity, and promising results have been reported [17,21,22]. Through this method, probiotics would remain viable and proliferate in zooplankton, thus ensuring effective administration in fish [23,24]. Probiotics could uplift the nutritional profile of zooplankton, and the positive effects of probiotics on fish health have been demonstrated by increasing the growth and survival of the host [21]. In addition, direct administration of probiotics to the culture water is risky due to easy exposure to microbiological contamination [21] and the short survival period of probiotics in seawater [25]. To our knowledge, however, no information is available regarding the application of probiotics to tilapia larvae via cladoceran *M. micrura*, even though they are commonly used as food for larval and post-larval rearing of freshwater crustaceans and teleost fish [26,27]. Thus, this study was designed to evaluate the efficacy of *M. micrura* as a possible vector of *B. pocheonensis* to enhance resistance to disease in red hybrid tilapia larvae.

## 2. Results

### 2.1. Antagonistic Activity of Bacillus pocheonensis S2 against Freshwater Pathogens

*Bacillus pocheonensis* was able to inhibit the growth of *S. agalactiae* at $10^{5}\;\mathrm{CFU}\;{\mathrm{mL}}^{-1}$ only. Meanwhile, *B. pocheonensis* was able to inhibit the growth of *A. hydrophila* at all three tested concentrations. The biggest inhibition zone was produced by probiont when tested against *A. hydrophila* at $10^{6}\;\mathrm{CFU}\;{\mathrm{mL}}^{-1}$ (Table 1).

### 2.2. Survival of Moina micrura after Challenged against Freshwater Pathogens

**Figure 1:**
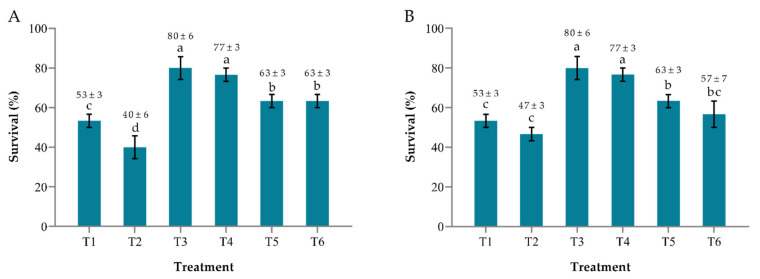
The survival percentage of *Moina micrura* after being treated with *Bacillus pocheonensis* at different concentrations and challenged with (**A**) *Streptococcus agalactiae* and (**B**) *Aeromonas hydrophila* at $10^{5}\;\mathrm{CFU}\;{\mathrm{mL}}^{-1}$. T1 (*M. micrura + C. vulgaris*); T2 (*M. micrura + C. vulgaris +* pathogen); T3 (*M. micrura* + *C. vulgaris* + *B. pocheonensis* at $10^{4}\;\mathrm{CFU}\;{\mathrm{mL}}^{-1}$); T4 (*M. micrura* + *C. vulgaris* + *B. pocheonensis* at $10^{6}\;\mathrm{CFU}\;{\mathrm{mL}}^{-1}$); T5 (*M. micrura* + *C. vulgaris* + *B. pocheonensis* at $10^{4}\;\mathrm{CFU}\;{\mathrm{mL}}^{-1}$ + pathogen); T6 (*M. micrura* + *C. vulgaris* + *B. pocheonensis* at $10^{6}\;\mathrm{CFU}\;{\mathrm{mL}}^{-1}$ + pathogen). Each value is the mean ± SEM of triplicate analysis. ^a,b,bc,c,d^ bars and mean with different alphabetical letters indicate a statistically significant difference (One-way ANOVA, *p* < 0.05).

In *S. agalactiae* challenged assay, the survival rates of *M. micrura* treated with *B. pocheonensis* at $5\times 10^{4}\;\mathrm{CFU}\;{\mathrm{mL}}^{-1}$ in treatment T5 and $5\times 10^{6}\;\mathrm{CFU}\;{\mathrm{mL}}^{-1}$ in treatment T6 were significantly higher (*p* < 0.05) than the control group in treatment T2 (Figure 1 A). These results suggested that *B. pocheonensis* at $5\times 10^{4}$ and $5\times 10^{6}\;\mathrm{CFU}\;{\mathrm{mL}}^{-1}$ were able to confer significant protection to *M. micrura* against *S. agalactiae* at $10^{5}\;\mathrm{CFU}\;{\mathrm{mL}}^{-1}$.

In *A. hydrophila* challenged assay, the survival rate of *M. micrura* treated with *B. pocheonensis* at $5\times 10^{4}\;\mathrm{CFU}\;{\mathrm{mL}}^{-1}$ in treatment T5 was significantly higher (*p* < 0.05) than the control group in treatment T2 (Figure 1B). Similarly, the survival rate of *M. micrura* treated with *B. pocheonensis* at $5\times 10^{6}\;\mathrm{CFU}\;{\mathrm{mL}}^{-1}$ in treatment T6 was higher than the control group in treatment T2, but they are not significantly different (*p* > 0.05). These results suggested that *B. pocheonensis* was able to provide significant protection to *M. micrura* against *A. hydrophila* at $5\times 10^{6}\;\mathrm{CFU}\;{\mathrm{mL}}^{-1}$ only.

### 2.3. Relative Percentage Survival (RPS) of Moina micrura after Challenged against Freshwater Pathogens

In *M. micrura* bacterial challenge tests, probiont-enriched *M. micrura* and unenriched *M. micrura* were challenged against pathogens *S. agalactiae* and *A. hydrophila*. The mortality of *M. micrura* in the control group started 24 h after being challenged and the average cumulative mortality was highest after 72 h post-challenge. Meanwhile, the mortality of *M. micrura* in both probiont-treated groups (T5 and T6) for both experiments started 48 h after being challenged.

In the *S. agalactiae* challenge experiment, the average cumulative mortalities of *M. micrura* in both treatments (T5 and T6) were significantly lower (*p* < 0.05) than the control group after 72 h post-challenge. The RPS of *M. micrura* in both probiont-treated groups was recorded at 38.33 (Table 2).

Meanwhile, in *A. hydrophila* challenge experiment, the average cumulative mortalities of *M. micrura* in T5 and T6 were lower than in the control group after 72 h post-challenge. Only *M. micrura* in treatment T5 had a significantly lower (*p* < 0.05) average cumulative mortality than the control group. The RPS of *M. micrura* in T5 and T6 was recorded at 30.19 and 18.87, respectively (Table 2).

### 2.4. Survival and Relative Percentage Survival (RPS) of Red Hybrid Tilapia Larvae

**Figure 2:**
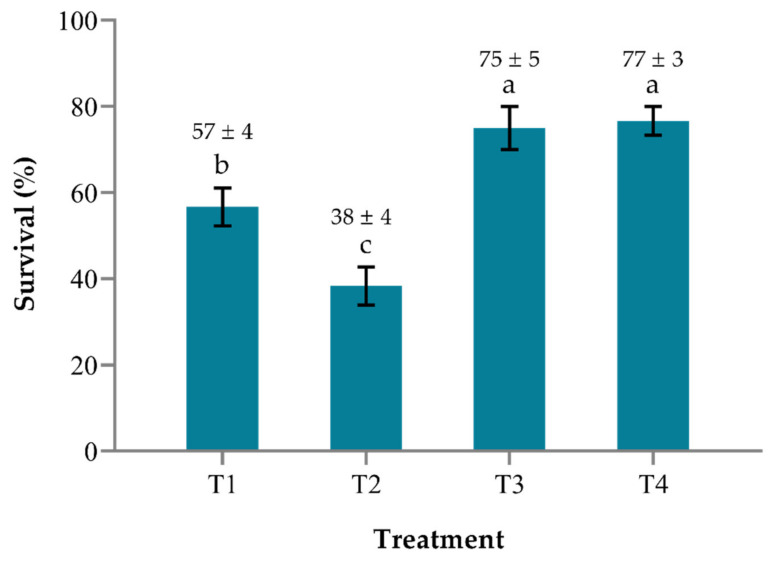
Survival of tilapia larvae fed with *Moina micrura* pre-enriched with *Bacillus pocheonensis* after challenged with *Streptococcus agalactiae*. T1(Tilapia + *M. micrura*), T2 (Tilapia + *M. micrura* + *S. agalactiae*), T3 (Tilapia + *M. micrura* + *B. pocheonensis*), T4 (Tilapia + *M. micrura* + *B. pocheonensis* + *S. agalactiae*). Each value is the mean ± SEM of triplicate analysis. ^a,b,c^ bars and mean with different alphabetical letters indicate a statistically significant difference (One-way ANOVA, *p* < 0.05).

The bacterial challenge assay lasted for 10 days and the mortality of tilapia larvae was first observed in the control group (T2) five days after the challenge. The administration of potential probiont *B. pocheonensis* at $5\times 10^{6}\;\mathrm{CFU}\;{\mathrm{mL}}^{-1}$ via *M. micrura* as vectors was able to confer significant protection to tilapia larvae against *S. agalactiae* at $10^{7}\;\mathrm{CFU}\;{\mathrm{mL}}^{-1}$ (Figure 2). Unchallenged tilapia larvae fed with probiont-enriched *M. micrura* in treatment T3 had a significantly higher (*p* < 0.05) survival rate than those in treatment T1 (no probiont), indicating that *B. pocheonensis* had no negative effect on tilapia larvae and may have improved tilapia health by increasing survival. Challenged tilapia larvae fed with probiont-enriched *M. micrura* in treatment T4 had a significantly higher (*p* < 0.05) survival rate than those in the control group in treatment T2, suggesting that *B. pocheonensis* may be beneficial to the host by enhancing disease resistance.

The mortality of tilapia larvae fed with unenriched *M. micrura* in the control group (T2) started on day 5 after being challenged. The average cumulative mortality was highest after 10 days of the experiment. The mortality of tilapia larvae fed with probiont-enriched *M. micrura* (T4) started on day 6 after being challenged. The average cumulative mortality was significantly lower (*p* < 0.05) than the control group after 10 days of the experiment. The RPS of tilapia larvae in the probiont-enriched group (T4) was recorded at 62.90 (Table 3).

### 2.5. Streptococcus agalactiae Counts

**Figure 3:**
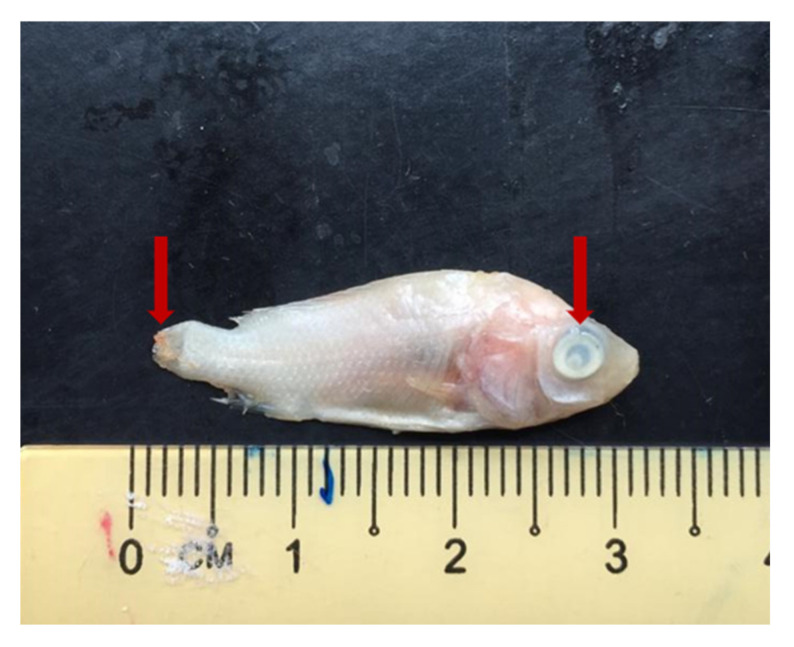
The external clinical signs observed on tilapia larvae after being challenged with *Streptococcus agalactiae* in the control group (T2) include cornea opacity and caudal fin erosion, as indicated by the red arrows.

The bacterial counts’ study revealed that potential probiont *B. pocheonensis* could partially reduce the concentration of *S. agalactiae* in tilapia larvae. The CFU of *S. agalactiae* in tilapia larvae fed with probiont-enriched *M. micrura* in treatment T4 was lower than those fed with unenriched *M. micrura* in treatment T2 (Table 4), but they are not significantly different (*p* > 0.05). In addition, some of the external clinical signs observed in tilapia larvae include spinning near the water surface, cornea opacity, and caudal fin erosion (Figure 3).

## 3. Discussion

The most frequently used probiotic bacteria in aquaculture practice belong to the genus *Bacillus* [28]. *Bacillus* spp. can facilitate digestive processes, modulate the immune system, and inhibit the growth of pathogens [29,30,31]. In addition, *Bacillus* spp. are commonly utilized for their sporulation capacity, non-pathogenic and non-toxic characteristics, and ability to produce antimicrobial substances [31]. In the present study, the 16S rRNA gene sequence analysis revealed that probiont showed 100% similarity with *B. pocheonensis*. *Bacillus pocheonensis* is a halotolerant bacterium first isolated from a soil sample of a ginseng field in Pocheon Province, South Korea [32]. However, there are a lack of studies reported on the probiotic properties of this species in reference to aquaculture practice.

The antagonistic activity against a variety of pathogens is one of the most important properties of probiotic candidates. In the present study, the antagonistic activity of *B. pocheonensis* against *S. agalactiae* and *A. hydrophila* at three different concentrations was evaluated in in vitro well-diffusion assay. The study revealed that *B. pocheonensis* antagonized *S. agalactiae* and *A. hydrophila* at least at one concentration out of the three. The inhibitory effect of *B. pocheonensis* against *S. agalactiae* at $10^{5}\;\mathrm{CFU}\;{\mathrm{mL}}^{-1}$ was the strongest, as indicated by the largest inhibition zone amongst treatments. Similarly, several studies have demonstrated the antagonism of *Bacillus* spp. isolated from various sources against a wide range of Gram-positive and Gram-negative pathogens, including *Vibrio parahaemolyticus*, *Vibrio campbellii*, and *Vibrio alginolyticus*, by means of secondary metabolites production, including siderophores, antibiotics, bacteriocins, and hydrolytic enzymes [33,34,35,36]. Several studies suggest that the production of bacteriocins and the inhibitory effects of *Bacillus* are likely due to the alteration of pH in the growth medium, utilization of essential nutrients, or production of volatile compounds [37,38,39]. 

In the present study, *M. micrura* was used as the host in preliminary in vivo bacterial challenge tests. The enrichment of *M. micrura* with *B. pocheonensis* at $10^{4}$ and $10^{6}\;\mathrm{CFU}\;{\mathrm{mL}}^{-1}$ was able to enhance the survival of *M. micrura* against *S. agalactiae* significantly. Meanwhile, *B. pocheonensis* was able to confer significant protection to *M. micrura* against *A. hydrophila* at $10^{4}\;\mathrm{CFU}\;{\mathrm{mL}}^{-1}$ only. The mechanisms by which the probiont confer protection against pathogen remain poorly understood, particularly in *M. micrura*. In the *Artemia* model study, enrichment with a single strain of probiotic bacteria at $10^{5}\;\mathrm{CFU}\;{\mathrm{mL}}^{-1}$ was able to protect *Artemia* from *Vibrio anguillarum* infection [40]. Additionally, the survival of *Artemia* was not significantly affected regardless of the species of probiotic bacteria used and the concentration applied. Similar to the present study, several published studies have suggested that low concentrations of probiotics ranging from $10^{4}$ to $10^{6}\;\mathrm{CFU}\;{\mathrm{mL}}^{-1}$ should be used in live feeds enrichment practice [40,41].

The administration of probiotics to fish and crustacean larvae via live feeds, such as *Artemia* and rotifers, has increasingly gained interest to improve the growth, survival, and disease resistance of fish larvae. In the present study, *Bacillus* sp. was administered via *M. micrura* to red hybrid tilapia larvae. Significant survival improvement of larvae fed with probiont-enriched *M. micrura* for 10 days was observed. Similarly, several published studies have reported that the administration of probiotics via live feeds enhanced the survival of fish and crustacean larvae, such as ornamental fish (*Poecilia latipinna*), European seabass (*Dicentrarchus labrax*), and Indian white shrimp (*Fenneropenaeus indicus*), after being challenged with freshwater and marine bacterial pathogens [42,43,44]. Survival and disease resistance enhancement is indeed an important feature of probiotic candidates.

Although the beneficial effect of probiotic application in enhancing the survival and disease resistance of fish larvae has been widely reported, the exact mechanisms by which probiotics promote survival are poorly understood. In the present study, the bacterial pathogen was introduced into tilapia larvae through the bath immersion method. Meanwhile in another study, the incorporation of *Bacillus velezensis* in the diet significantly increased tilapia survival after being challenged with *S. agalactiae* by intraperitoneal (IP) injection [45]. Merrifield et al. [46] suggested that the ability of probiotics to prevent disease may be greater than the results reported in many studies that employed the IP injection method in challenge studies. The IP injection method bypasses competitive exclusion, one of the most important mechanisms in which probiotics prevent pathogen infection in the gastrointestinal tract (GI) [47]. In addition, as the GI tract of most fish species at the early larval stage is poorly developed, the establishment of the microbial community is influenced by the aquatic environments and live feeds [21]. Thus, the administration of probiotics should be made early and frequently to establish the artificial dominance of beneficial bacteria [48].

Probiotics were demonstrated to have the efficacy to inhibit the growth of pathogens in vivo. In the present study, enrichment of *M. micrura* with *B. pocheonensis* at $5\times 10^{6}\;\mathrm{CFU}\;{\mathrm{mL}}^{-1}$ for 3 h by immersion was able to partially reduce the concentration of *S. agalactiae* in tilapia larvae. Similar results are observed for juvenile blue swimming crab (*Portunus pelagicus*) supplemented with *Bacillus amyloliquefaciens* and challenged with *Vibrio harveyi* [49]. Similarly, the enrichment of copepod with *Bacillus clausii* or *Bacillus pumilus* at $10^{6}\;\mathrm{CFU}\;{\mathrm{mL}}^{-1}$ for 3 h by immersion was able to reduce the concentration of pathogen *Aliivibrio fischeri* and *Vibrio* spp. in the gut of grouper (*Epinephelus coioides*) larvae [21]. In another study, the supplementation of *Bacillus subtilis* at $10^{8}$ and $10^{9}\;\mathrm{CFU}\;L^{-1}$ to the rearing water of white shrimp (*Litopenaeus vannamei*) larvae were able to significantly reduce the concentration of presumptive *Vibrio* spp. [50].

The competition for adhesion sites through competitive exclusion has been widely proposed to explain the reduction of pathogenic bacteria in the fish gut [51]. The present study suggested that the supplementation of *B. pocheonensis* was effective in reducing the *S. agalactiae* load in tilapia larvae. The action mechanism of *B. pocheonensis* being based on competitive exclusion seems like a plausible explanation due to the retrieval of *B. pocheonensis* in challenged tilapia larvae at the end of the experiment. Xia et al. [52] agreed that the changes in the composition of the gut bacterial community and the enhancement of the composition of some beneficial bacteria in the gut may have influenced tilapia resistance to streptococcosis. Successful colonization of the fish gut by probiotic bacteria is indeed difficult to achieve [53]. Factors, including the selection of probiotic strain, probiotic dosage, modes of probiotic administration, and frequency of probiotic supplementation may influence the colonization success [52,54].

## 4. Materials and Methods

### 4.1. Bacterial Strains and Growth Conditions

*Bacillus pocheonensis* strain S2 (GenBank accession no. MK764898) was previously isolated from microalgae *Spirulina* sp. [55,56]. *Aeromonas hydrophila* and *S. agalactiae* were obtained from Fish Health Laboratory, Faculty of Agriculture, Universiti Putra Malaysia (UPM), Malaysia. All three bacteria were cultured in trypticase soy broth (TSB) (Merck, Darmstadt, Germany) and incubated overnight at 30 °C with constant shaking at 150 rpm (Biosan, Riga, Latvia). On the next day, all cultures were centrifuged (Hermle, Wehingen, Germany) at 5000× *g* for 10 min at room temperature. The supernatants were discarded, and the pellets were resuspended in sterile distilled water and washed once. The concentrations of all cultures were determined using a spectrophotometer (Eppendorf, Hamburg, Germany) at the optical density of 550 nm $\left({\mathrm{OD}}_{550}\right)$. The concentration of all bacteria was then adjusted accordingly to the desired sufficient value $\left(\;\mathrm{CFU}\;{\mathrm{mL}}^{-1}\right)$.

### 4.2. Cultures of Microalgae Chlorella vulgaris

Microalgae *C. vulgaris* (Isolate number: UPMC-A0088) was obtained from Aquatic Animal Health and Therapeutics Laboratory (AquaHealth), Institute of Bioscience (IBS), UPM, Malaysia. *Chlorella vulgaris* cultures were grown in sterile 1000 mL conical flasks, filled with Bold’s Basal medium (BBM) with constant shaking at 60 rpm on an orbital shaker (Protech, Balakong, Malaysia) and continuous light. The concentration of *C. vulgaris* was determined using a 0.1 mm improved Neubauer chamber observed under a light microscope according to the following formula:

$$\mathrm{Density},\;d\;(\mathrm{cells}/\mathrm{mL})=\frac{\mathrm{Average}\;\mathrm{number}\;\mathrm{of}\;\mathrm{cells}\;\mathrm{per}\;\mathrm{square}\;\mathrm{counted}}{4\times 10^{-6}}$$

where $4\times 10^{-6}$ = the volume of sample over the small square area, which is equivalent to $0.004{\mathrm{mm}}^{3}$ (0.2 × 0.2 × 0.1) expressed in ${\mathrm{cm}}^{3}$ (mL).

### 4.3. Preliminary In Vitro Screening of Bacillus pocheonensis S2

Agar well-diffusion method was used to screen the antagonistic activity of *B. pocheonensis* against freshwater pathogens *S. agalactiae* and *A. hydrophila.* The concentrations of both pathogens were adjusted accordingly. The trypticase soy agar (TSA) (Defco Merck, Germany) plates were aseptically inoculated by individually swabbing each pathogen at the final concentration of $5\times 10^{5}$, $5\times 10^{6}$, and $5\times 10^{7}\;\mathrm{CFU}\;{\mathrm{mL}}^{-1}$ over the entire agar surface using a sterile cotton swab. Holes with a diameter of 4 mm were aseptically punched on each TSA plate using a sterile cork borer. Then, 10 µL probiont culture at the final concentration of $10^{9}\;\mathrm{CFU}\;{\mathrm{mL}}^{-1}$ was dispensed into each well. All plates were incubated overnight at 30 °C. On the next day, the inhibition zone was observed, and the size was measured.

### 4.4. Culture and Maintenance of Moina micrura

*Moina micrura* was obtained from the AquaHealth Laboratory, IBS, UPM, Malaysia. *Moina micrura* was maintained in 1000 mL plastic aquaria filled with filter-sterilized freshwater pond water (0.45 µm fiberglass filters) with mild aeration. The water media was exchanged once a week. *Moina* was fed with microalgae *C. vulgaris ad libitum* [57,58,59].

### 4.5. Preliminary In Vivo Bacterial Challenge Assay of Moina micrura

This method is based on Masduki et al. [60] with minor modifications. Ten *M. micrura* were placed in a 50 mL Falcon tube containing 30 mL sterilized freshwater pond water. *Moina micrura* was first pre-incubated with probiont *B. pocheonensis* at two different concentrations: $5\times 10^{4}$ and $5\times 10^{6}\;\mathrm{CFU}\;{\mathrm{mL}}^{-1}$ for 24 h. *Moina* was fed with *C. vulgaris* at $4\times 10^{5}\;\mathrm{cells}\;{\mathrm{mL}}^{-1}$ once a day. After 24 h pre-incubation, pathogens *S. agalactiae* and *A. hydrophila* at $5\times 10^{5}\;\mathrm{CFU}\;{\mathrm{mL}}^{-1}$ were individually introduced into the culture. All tubes were incubated at room temperature at a normal condition of 12 h light and 12 h dark regime. The observation was made until the mortality of *M. micrura* in the control group (without probiont) reached at least 50%. The survival rate of *M. micrura* was determined as below:

(1) $$\mathrm{Survival}\;\mathrm{rate}\;\left(\%\right)=(\frac{\mathrm{Number}\;\mathrm{of}\;\mathrm{survivors}}{\mathrm{Total}\;\mathrm{number}\;\mathrm{of}\;\mathrm{larvae}})\times 100$$

The efficacy of the potential probiont was assessed by calculating the relative percentage survival (RPS). The RPS was calculated using the formula:

(2) $$\mathrm{RPS}=1-(\frac{\%\mathrm{mortality}\;\mathrm{in}\;\mathrm{test}\;\mathrm{group}}{\%\mathrm{mortality}\;\mathrm{in}\;\mathrm{control}\;\mathrm{group}})\times 100$$

### 4.6. Enrichment of Moina micrura

*Moina micrura* was cultured in 5 L plastic aquariums filled with sterilized freshwater pond water. *Moina micrura* was fed with *C. vulgaris* cultured in BBM ad libitum at $4\times 10^{5}\;\mathrm{cells}\;{\mathrm{mL}}^{-1}$ [61]. An air pump was used to provide mild aeration. Before probiont enrichment, *M. micrura* was harvested early in the morning using a 200 µm sieve and thoroughly washed with tap water. *Moina micrura* was concentrated in 2 L plastic aquaria filled with sterilized freshwater pond water, at approximately $10^{5}\;Moina\;L^{-1}$. Cultures of *M. micrura* to be fed to probiont-treated tilapia larvae were inoculated with *B. pocheonensis* at $5\times 10^{6}\;\mathrm{CFU}\;{\mathrm{mL}}^{-1}$ and incubated for 3 h [21] at room temperature with mild aeration.

### 4.7. Experimental Design

This method is based on Sun et al. [21] with a minor modification on the number of tilapia larvae used, live feed density, and survival determination. Each 10 L plastic tank filled with 5 L tap water was randomly stocked with 20 tilapia larvae with an average weight of 0.34 g and 25 mm in length. Treated tilapia larvae were fed with *M. micrura* pre-enriched with potential probiont *B. pocheonensis* at $10^{6}\;\mathrm{CFU}\;{\mathrm{mL}}^{-1}$. Meanwhile, untreated tilapia larvae were fed with control *M. micrura* (without *B. pocheonensis* enrichment). Both treated and untreated *M. micrura* were fed to tilapia larvae once a day at a density of approximately 2.0 *Moina* ${\mathrm{mL}}^{-1}$. Each treatment was carried out in triplicate.

After the first 24 h, pathogenic *S. agalactiae* was inoculated in treatment T2 and T4 at $10^{7}\;\mathrm{CFU}\;{\mathrm{mL}}^{-1}$ for a bacterial challenge test. Tilapia larvae in tanks with no pathogen inoculation served as survival control, while those in tanks with pathogen inoculation alone served as mortality control. Treated tilapia larvae fed with *M. micrura* enriched with *B. pocheonensis* served as probiotic control. Meanwhile, larvae fed with *M. micrura* enriched with *B. pocheonensis* and inoculated with *S. agalactiae* served as the experimental treatment group. All tanks were provided with continuous aeration and the water temperature was maintained at 28 °C. The challenge test was carried out until tilapia larvae in the mortality control group recorded at least 50% mortality.

The survival of tilapia larvae was determined by counting the number of larvae remaining in each tank when the mortality control group recorded at least 50% mortality. The survival rates and RPS of red tilapia larvae were determined using the formulae previously described.

### 4.8. Streptococcus agalactiae Counts

A total plate count of *S. agalactiae* was carried out to evaluate the ability of potential probiont *B. pocheonensis* administered via *M. micrura* in inhibiting the growth of *S. agalactiae* in tilapia larvae. After counting the number of living and dead larvae, the tilapia larvae from treatments T2 and T4 were collected at the end of the experiment. The larvae were collected and washed with sterilized distilled water. The tilapia larvae were homogenized using sterile porcelain mortar and pestle. A serial dilution of the homogenated larvae was made up to $10^{-6}$ in sterilized distilled water. Following serial dilution, 100 µL of each diluted sample was pipetted and dispensed onto Brain Heart Infusion (BHI) (Merck, Darmstadt, Germany) agar medium. The samples were spread evenly onto BHI agar using a sterile glass hockey stick. Each sample was plated out in triplicates. The plates were incubated overnight at 30 °C and the colony-forming unit (CFU) was counted on the following day. The confirmation of *S. agalactiae* isolate was determined by performing Gram staining and 16S rRNA gene analysis. In addition, throughout the experiment, the clinical signs of *S. agalactiae* infections in tilapia larvae were observed.

### 4.9. Statistical Analysis

All the collected data were analyzed using one-way analysis of variance (ANOVA) and the Tukey test was carried out for multiple comparison tests. Significant difference was at *p* < 0.05. All statistical tests were performed using GraphPad Prism 8 (GraphPad Inc., San Diego, CA, USA). 

## 5. Conclusions

The intensification of culture systems to meet the increasing global demand for fish as a healthy source of animal protein has brought about the application of eco-friendly feed additives, such as probiotics, as a means of disease control and prevention. The present study evaluated the efficacy of *B. pocheonensis* administered to red tilapia larvae via *M. micrura*. Our results demonstrated the ability of *B. pocheonensis* to positively influence *M. micrura* and larval survival after being challenged with *S. agalactiae*. The reduction of *S. agalactiae* load and recovery of *B. pocheonensis* in tilapia larvae after being challenged may be related to the improvement of survival and health of larvae. Therefore, the results in our study suggested that the application of *M. micrura* is very much suitable to function as vectors for probiotics administration to red hybrid tilapia larvae. Moreover, this probiotic–zooplankton coupling has a high potential in eliminating or reducing the need for antibiotic usage in aquaculture industries. Although the results in our study present evidence that *B. pocheonensis* is capable of enhancing disease resistance in larval red hybrid tilapia, the specific mode of actions remain unclear and merit further research. Furthermore, the present study encourages further research on the intestinal microbiota profiling of larval red hybrid tilapia and the retention period of probiotic inocula it receives.

## Figures and Tables

**Table 1:** Diameter of inhibition zones by *Bacillus pocheonensis* against *Aeromonas hydrophila* (AH) and *Streptococcus agalactiae* (SA) at three concentrations.

Pathogens	Concentration $\left(\;\mathrm{CFU}\;{\mathrm{mL}}^{-1}\right)$	Diameter of Inhibition Zone (mm)
SA	$10^{5}$	18.2 ± 0.4
	$10^{6}$	-
	$10^{7}$	-
AH	$10^{5}$	10.7 ± 0.3
	$10^{6}$	11.0 ± 0.0
	$10^{7}$	8.8 ± 0.2

Size of inhibition zone ± SEM; (-) = No clear zone was observed.

**Table 2:** Mortality (%) and relative percentage survival (RPS) of *Moina micrura* treated with *Bacillus pocheonensis* after 72 h post-challenge with *Streptococcus agalactiae* (SA) and *Aeromonas hydrophila* (AH).

Treatment (T)	Descriptions	Average Mortality	RPS
2	*M. micrura* + *C. vulgaris* + SA	60±6 ^a^	-
5	*M. micrura* + *C. vulgaris* + *B. pocheonensis* at $10^{4}\;\mathrm{CFU}\;{\mathrm{mL}}^{-1}$ + SA	37±3 ^b^	38.33
6	*M. micrura* + *C. vulgaris* + *B. pocheonensis* at $10^{6}\;\mathrm{CFU}\;{\mathrm{mL}}^{-1}$ + SA	37±3 ^b^	38.33
2	*M. micrura* + *C. vulgaris* + AH	53±3 ^a^	-
5	*M. micrura* + *C. vulgaris* + *B. pocheonensis* at $10^{4}\;\mathrm{CFU}\;{\mathrm{mL}}^{-1}$ + AH	37±3 ^b^	30.19
6	*M. micrura* + *C. vulgaris* + *B. pocheonensis* at $10^{6}\;\mathrm{CFU}\;{\mathrm{mL}}^{-1}$ + AH	43±7 ^ab^	18.87

Each mortality value is the mean ± SEM of triplicate analysis. +, composition of each treatment. ^a,b,ab^ mean with different alphabetical letters indicate a statistically significant difference (*p* < 0.05).

**Table 3:** Mortality (%) and relative percentage survival (RPS) of tilapia larvae after challenged with *Streptococcus agalactiae* following 10 days of the experiment.

Treatment (T)	Descriptions	Average Mortality	RPS
2	Tilapia + *M. micrura* + *S. agalactiae*	62±4 ^a^	-
4	Tilapia + *M. micrura* + *B. pocheonensis* + *S. agalactiae*	23±3 ^b^	62.90

Each mortality value is the mean ± SEM of triplicate analysis. +, composition of each treatment. ^a,b^ mean with different alphabetical letters indicate a statistically significant difference (*p* < 0.05).

**Table 4:** Bacterial counts on tilapia larvae fed with unenriched and enriched *Moina micrura* after challenged *with Streptococcus agalactiae*.

Treatment (T)	Descriptions	$\mathrm{Log}\;10\;\mathrm{CFU}\;{\mathrm{mL}}^{-1}$
2	Tilapia + *M. micrura* + *S. agalactiae*	7.78±0.09 ^a^
4	Tilapia + *M. micrura* + *B. pocheonensis* + *S. agalactiae*	6.84±0.39 ^a^

Each value is the mean ± SEM of triplicate analysis. +, composition of each treatment. ^a^ The same superscript indicates no significant difference (*p* > 0.05).

## Data Availability

All data generated or analyzed during this study have been included in this article.
